# Telehealth Consultation for Malaysian Citizens’ Willingness to Pay Assessed by the Double-Bounded Dichotomous Choice Method

**DOI:** 10.21315/mjms2024.31.1.8

**Published:** 2024-02-28

**Authors:** Tan Mey Mey, Katsuhiko Ogasawara

**Affiliations:** Graduate School of Health Sciences, School of Medicine, Hokkaido University, Hokkaido, Japan

**Keywords:** telehealth, contingent valuation methods, Double-Bounded Dichotomous Choice, willingness to pay

## Abstract

**Background:**

Re-envisioning healthcare in technology tools includes robust utilisation of telehealth, improvement in access, quality, care efficiencies and cost-effectiveness of healthcare services. In reality, the technology’s potential to transform healthcare may be limited by the ability to pay for it. This study aims to estimate Malaysian citizens’ willingness to pay (WTP) for telehealth consultations and determine the factors contributing to it. This is vital to inform decision-making about expansion, preferences and deployment of a pricing strategy for telehealth services.

**Methods:**

A random sample of 220 adult Malaysians was surveyed using social network services (SNS). Three different WTP bid arrays were identified and each respondent received a randomly drawn bid price. The WTP fee for using the telehealth consultation for 30 min was measured and estimated using a Double-Bounded Dichotomous Choice (DBDC) and the Random Utility Logit Model.

**Result:**

The median WTP was estimated to be RM58 (JPY2,198), RM78 (JPY2,956) for 132 respondents’ willingness to use telehealth consultation and RM26 (JPY985) for 51 respondents who were unwilling. Further analysis found that WTP is correlated with the perception and willingness of the respondents to use it.

**Conclusion:**

Despite most respondents being willing to pay for telehealth consultations, sociodemographic characteristics and affordability influenced the process of making decisions about WTP for telehealth consultation. This finding suggests that the private sector can play a crucial role in the deployment of telehealth. However, there may be a need to consider affordability and how to increase access and use of telehealth services.

## Introduction

Escalating healthcare expenditures, the introduction of expensive medical technology and intensification of consumer demand necessitate healthcare reforms (1). Healthcare reforms typically attempt to expand the population that receives healthcare through public sector insurance schemes or private sector insurers, expand the range of healthcare providers available to consumers, improve access to healthcare specialists, improve the quality of healthcare and ultimately reduce the cost of healthcare (2). These reforms have also promoted increasingly cost-conscious healthcare practices (3), consequently encouraging new trends in medical device innovation, such as telehealth. Innovations in e-health technology development have led to a simultaneous expectation that they will lead to significant efficiency gains and reduction in overall health expenditure (4). In addition, the successful innovation of electronic health boards brings new changes in technology and applications that will facilitate clinical decision-making, improve the quality and efficiency of care, engage individuals in clinical decision-making and enable patients to adopt healthy behaviour (5). Furthermore, telehealth demonstrates enormous potential for increased productivity from health system redesign by offering overwhelmingly positive patient benefits, potential for cost savings and opportunity to develop culturally appropriate services that are more sensitive to the needs of special populations (6). Thus, telehealth is emerging as a critical component of the solution to the healthcare crisis (7).

In Malaysia the terms ‘telemedicine,’ ‘telehealth’ and ‘e-health’ are often used interchangeably, although telehealth is sometimes used more broadly for remote health not involving active clinical treatments. The Malaysian government has implemented the Telemedicine Act since 1997 to strengthen and reshape healthcare services via the utilisation of digital health technologies to improve the efficiency of healthcare services (8). However, the growth in telehealth adoption was a major challenge, even before the spread of the global COVID-19 pandemic. Telehealth in Malaysia is still developing in the context of a dual-tiered healthcare delivery system comprising a heavily subsidised public sector from the government and thriving parallel fee-for-service private healthcare system (9). While telehealth consultation services have only recently been established in some hospitals, other telehealth companies have struggled for months or even years to embark on digital service delivery for healthcare. Innovative e-health technologies are crucial to enhance healthcare accessibility in Malaysia and ensure a vision of quality, affordable, and patient-centered healthcare.

A study by the Ministry of Health Malaysia found that the average distance to hospitals in rural areas is more than twice that of urban hospitals, 36 km for rural residents and 17 km for urban residents (10). Residents of rural areas often travel long distances over rough terrain to reach medical facilities. Therefore, these disparities in distance and travel time hinder access to healthcare services for the rural population of Malaysia. The differences between East Malaysia and Peninsular Malaysia in terms of population density, accessibility and types of services available have become an obstacle to delivering healthcare services to rural communities (11). A study by Ng et al. (12) found that less than half of primary care clinics in Malaysia provided telehealth consultation and a majority of the clinics providing telehealth consultation were in urban areas. Discrepancy in the availability of telehealth consultation between demographics and regions could be attributed to the digital divide, which is giving rise to inequalities in access to opportunities, knowledge and services in healthcare (13).

Telehealth strategies are conceived as a tool to reshape the healthcare delivery system by simultaneously increasing access to healthcare services, improving efficiency and reducing overall health spending (14). Conversely, the expansion of telehealth may exacerbate existing health disparities by creating digital divide inequalities in access to information and communication technologies, thereby having a greater impact on health inequalities driven by socioeconomic determinants of health. There is a consideration threat that, while the COVID-19 pandemic has unleashed unprecedented massive acceleration in the use of digital health services, it has also exposed the inadequacies of the private fee-for-service model of healthcare that has primarily relied on out-of-pocket costs. Telehealth usage varies significantly by different social demographic groups and income disparities (15); these inequalities in access and usage should be monitored in order to truly close the healthcare gap. A recent study at the University of California, San Francisco found inequities in the use and access to telehealth during the COVID-19 pandemic; more than one-third of adults over the age of 65 years old faced potential difficulties in meeting a doctor via telehealth, with older, low-income men in remote or rural areas facing the greatest challenges. This is especially true for people with disabilities or medical conditions (16). In other words, policy changes have helped eliminate some barriers to telehealth access and have facilitated the expanded use of telehealth as an increasing number of medical service providers and clinics are equipped with the infrastructure required to provide telehealth services. This may also raise challenges in offering affordable and patient-centred healthcare on a routine basis in the future (17).

As telehealth gains momentum, more research outcomes and inputs on quality, convenience and cost from a patient’s perspective are needed (18). There are very few written studies on willingness to pay (WTP) for telehealth and none in Malaysia. Therefore, the aim of this research is to identify Malaysian citizens’ preferences for telehealth services and utilise a contingent valuation method to estimate their WTP.

Healthcare is the most regulated sector of the economy, therefore, extending telehealth services will require regulatory reform in addition to consumer demand. There has been limited exploration of the WTP from a response perspective, despite it being pertinent to the sustainability of telehealth interventions. Additionally, telehealth growth will also depend on WTP for telehealth visits in the future, as WTP also serves as a surrogate for the demand and acceptability of such services. In this regard, this research also identifies underlying factors influencing WTP and willingness to use telehealth services to suggest ways for policymakers and funders to determine whether the evidence supports wider adoption of telehealth interventions in Malaysia (19).

Taking into consideration one’s WTP, the buyer would evaluate whether the trade is beneficial for themselves and would subsequently make a purchase when their WTP is greater than the cost of the object sold. The outcomes not only provide relevant monetary estimates but can also be used as an indication of the intensity of the sample population’s interest in receiving telehealth services and likely service demand.

At present, the available telehealth consultations are mainly for follow-up patients and those who seek a second opinion (20). Most funding from the Ministry of Health’s public primary care clinics provides video consultations only at select clinics and there is limited information on the expansion and availability of telehealth consultations in Malaysian primary care institutions (21). This study estimated the contingent value of telehealth services by eliciting respondents’ preferences and placing a monetary value on the benefit associated with healthcare follow-up from a societal perspective. Telehealth consultation includes initial and follow-up appointments through private and government-provided online telehealth consultation services. As a concept, WTP is the maximum amount that an individual is willing to forgo in order to use or consume particular goods or services (22). This evidence-based research can also be used as a reference to help policymakers make decisions about pricing strategy, resource allocation and other economic decisions in scale and optimise telehealth programmes in Malaysia (23).

It is assumed that telehealth services in Malaysia rely on individual preferences and can be characterised by substitution between income and other specific factors. Consequently, it is hypothesised that WTP for telehealth consultation will vary according to the age and household income of the individual. Education is expected to affect willingness to pay, with higher levels of education associated with higher household income, as awareness and willingness to access healthcare information is likely to be greater.

## Methods

### Study Design Sampling and Respondents

In this study, a Double-Bounded Dichotomous Choice (DBDC) survey design with open-ended question format was used to elicit 20 years old–59 years old Malaysian citizens’ WTP for 30 min of telehealth consultation per session. The advantage of the double-bounded model is that it provides more information to better estimate an individual’s true WTP and produces less biased WTP estimates than the single-bounded model (24). Thus, DBDC is the most plausible choice for this research paper.

This survey was an online questionnaire distributed via a social network service (SNS) through a Google document format. It consisted of the following four parts: i) participant characteristics, ii) WTP, iii) perceptions of telehealth service/willingness to use and iv) satisfaction level. Based on a 95% confidence interval and 5% margin of error, the required study sample size was calculated as 384 using a single proportion formula. Three different SNSs were selected to ensure that the study was representative of the whole population of Malaysia. A total of 220 study respondents were successfully enrolled between April 2022 and June 2022. To gather information supporting the true WTP distribution, the starting bids were varied and bid vectors were chosen by considering the results of the pre-test pilot survey. As a result, three different WTP bid arrays were identified and modified using the contingent valuation format, and each respondent received a randomly drawn bid price.

### Data Analysis

In contingent valuation surveys, respondents are directly asked their maximum WTP by answering ‘yes’ or ‘no’ to whether they are WTP a given amount (bid) to be consulted by the same doctor via telehealth consultation instead of visiting a clinic for a follow-up. Respondents were asked to answer two consecutive questions.

If the respondents answered ‘yes’ to the initial bid then that amounted to *P_i_*, following which an iterative-bidding question with a higher amount *P_i_^U^* was shared. If a respondent answered ‘no’ to the first step, a lower bid *P_i_^L^* amount was presented in the second step. Therefore, *P_i_^L^* < *P_i_* < *P_i_^U^*. Thus, the DBDC format is statistically more efficient than the Single-Bounded Dichotomous Choice (SBDC) format and has a narrower confidence interval for the estimated WTP (25).

The response data was analysed using the logit model derived from the random utility model (26). The probability answers to the DBDC questions were interpreted in four patterns, ƒ^NN^, ƒ^YN^ and ƒ^NY^ as shown below. Formally, ƒ^YY^, ‘Yes’ to both the first and second question; ƒ^NN^, ‘No’ to both the first bid and second bid (NN); ƒ^YN^, ‘Yes’ to the first question and ‘No’ to the second bid; and ƒ^NY^, ‘No’ to the first bid question and ‘Yes’ to the second lower bid (NY). *P_i_* represents the probability of WTP for telehealth consultation, the distribution function is represented as G and *θ* is the parameter vector. Assuming that the responses are consistent, the four probabilities for each response can be presented as follows:


$$\begin{array}{l} f^{\text{YY}}(P_{i},{P_{i}}^{u})=\text{Tr}\{\text{WTP}\geq P_{i},\;\text{WTP}\geq {P_{i}}^{U}\}=1-\text{G}({P_{i}}^{U},\theta )\\ f^{\text{NN}}(P_{i},{P_{i}}^{L})=\text{Tr}\{\text{WTP}\leq P_{i},\;\text{WTP}\leq {P_{i}}^{L}\}=\text{G}({P_{i}}^{L},\theta )\\ f^{\text{YN}}(P_{i},{P_{i}}^{U})=\text{Tr}\{P_{i}\leq \text{WTP}\leq {P_{i}}^{U}\}=\text{G}({P_{i}}^{U};\theta )-\text{G}(P_{i};\theta )\\ f^{\text{NY}}({P_{i}}^{L},P_{i})=\text{Tr}\{P_{i}\geq \text{WTP}\geq {P_{i}}^{L}\}=\text{G}(P_{i};\theta )-\text{G}({P_{i}}^{L};\theta ) \end{array}$$

The log-likelihood function for independent observations is written as follows:


$${LnL}^{\text{D}}(\theta )=\Sigma_{i}^{N}\{{b_{i}}^{YY}{Inf}^{YY}(P_{i},{P_{i}}^{U})+{b_{i}}^{NN}{Inf}^{NN}(P_{i},{P_{i}}^{L})+{b_{i}}^{YN}{Inf}^{YN}(P_{i},{P_{i}}^{U})+{b_{i}}^{NY}{Inf}^{NY}(P_{i},{P_{i}}^{L})$$

where *b_i_^NN^*, *b_i_^YN^* and *b_i_^NY^* are dummy variables that correspond to each response pattern and *P_i_* is the bid for the respondents.

For example, *b_i_^YY^* is a variable assuming *b_i_^YY^* =1 if the respondent answers ‘Yes’ to both the first bids *P_i_* and the second bid *P_i_^U^* and *b_i_^NN^* = 0 otherwise.

Use a log-linear function for *P (i)* and the constant term *α* and the parameter of logarithmic value of bid *β* for *θ* as below:


$$Y(P)=\frac{1}{1+exp\left\{-\left(\alpha +\beta Inp\right)\right\}}$$

where *P* is the bid for the respondents. The median WTP can be calculated by using the estimated parameters *α* and *β* (27). The median indicates the probability that a respondent will answer ‘Yes’ to a bid with a probability of 0.5 and can be calculated as follows:


$$\text{Median WTP}=\text{exp }\left(-\frac{\alpha }{\beta }\right)$$

The mean WTP was obtained by integrating the probability of respondents answering ‘Yes’ in relation to their bids and is calculated as follows:


$$MeanWTP\;(trancation\;at\;T_{max}\int_{0}^{Tmax}\frac{1}{1+{exp}^{-\Delta V}}dt$$

where *T_max_* is the maximum bid. The analyses for this study were carried out using Koichi Kuriyama’s ‘CVM version 4.0’ (http://kkuri.eco.coocan.jp/). The results of respondents bidding on the DBDC model are shown in Table 1.

## Results

The survey results were *n* = 70 men and *n* = 150 women with a mean age of 40 years old–49 years old. There were more female respondents than male respondents (68% versus 32%). More than half of the respondents had a university degree (*n* = 175, 79.5%) and only 2.3% had primary education. Most respondents (*n* = 171, 78.1%) were employed, with 12 being self-employed. About 16.9% of the respondents were unemployed. Sixty respondents had a lower personal income level (< RM2,000 per month); of them, 37 were unemployed. Additionally, 56% of high-income respondents (RM6,000–RM9,000 and those with more than RM10,000) were significantly more willing to use these services. Table 2 shows the sociodemographic characteristics of the respondents.

**Figure 1:**
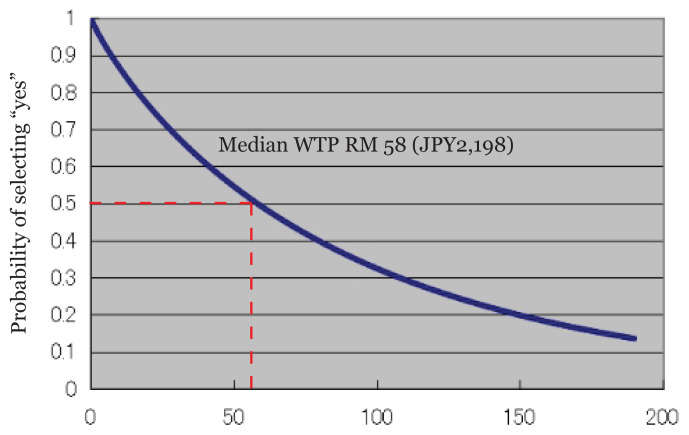
The estimated demand curve

The estimated median WTP for teleconsultation is RM58 (JPY2,198), as seen in Figure 1. Currently, in Malaysia, online health providers cite a fee of approximately RM20 (JPY758) for telehealth consultation with a general practitioner and RM40 (JPY1,516) for consultation with a specialist (28). In contrast, telehealth consultations provided by the private sector charge a minimum of RM70 (JPY2,653) per consultation for about 15 min to 20 min, with the charges varying according to different private hospitals (29).

## Discussion

### Factor Influencing Willingness to Pay

In addition to determining the WTP, it is essential to explore the factors influencing it and understand the preference (in terms of WTP) for telehealth services, as this may inform health policymakers for optimal resource allocation and pricing strategies for telehealth services (30). Table 2 shows that respondents’ sociodemographic variables including age, education level, household income and willingness to use had a significant impact on their WTP and were independently associated with the amount they were willing to pay for telehealth services in Malaysia.

Many studies suggest that WTP is influenced by several factors (31) and age is a known factor associated with information and communications technology (ICT) use and comfort, including the use of health information (32). To determine the effect of age on WTP in this study, the age distributions were adjusted and respondents were categorised into four age groups: i) 20 years old–29 years old, ii) 30 years old–39 years old, iii) 40 years old–49 years old and iv) 50 years old–59 years old. There was only a slight difference between the 30 years old–39 years old of age group with a WTP median of RM64 (JPY2,426) and the 50 years old–59 years old of age group with a WTP median of RM61 (JPY2,311). However, the adolescent/young adult generation (20 years old–29 years old) showed a higher median WTP of RM71 (JPY2,691). It is possible that the younger respondents and those with middle income levels had positive attitudes toward telehealth, as most of them were university graduates with a WTP of RM59 (JPY2,691). They tended to be more willing to engage in and adapt to digital and health technologies (33).

The results for those in the age group of 40 years old–49 years old, with a median WTP of RM43 (JPY1,630), was substantially lower than those for the other age groups. They preferred face-to-face in-person care, while only 37 out of 71 responses said they would use telehealth consultation when the COVID-19 pandemic was declared. Interestingly, of the 71 responses from this middle-aged group, 45 (63.3%) claimed that insurance companies should cover telehealth services. Clearly, reimbursement was one of the barriers to the adoption and use of telehealth (34). As evidenced from a survey by KLAS Research and Healthcare Information Management Executive Institute of 104 healthcare organisations about existing telehealth programmes, reimbursement scored highest (59%) among the other score barriers to telehealth expansion (35).

Among sociodemographic variables, age and income also showed significant effects on WTP responses. A report by Tan et al. (36) found that there is a direct correlation between ‘ability’ and ‘WTP’. The estimated median WTP for high-income respondents (more than RM10,000 per month) was RM107 (JPY4,055), for lowest income respondents (less than RM2,000 per month), median WTP was RM62 (JPY2,350). Therefore, the logarithm of monthly income influenced the WTP difference. Low income is a barrier to engaging in telehealth; it also affects the populations’ health, such as difficulty accessing care systems. In other words, respondents with lower income are less WTP for telehealth.

### The Older Generation

The elderly generation tends to have complex healthcare needs, and telehealth plays a vital role in improving their access to healthcare and reducing healthcare costs. However, technology adoption among older adults may pose challenges to the successful implementation of innovative telehealth services to reduce health disparities (37). Consequently, it is imperative to assess whether the elderly generation is generally WTP for telehealth consultation, which is an important prerequisite for the successful implementation of e-health interventions. However, this study did not include any respondents older than 69 years old. Future research should explore how WTP is driven by specific geriatric needs, the ability of technology to improve access to older adult care and affordable telehealth for the older population. Additionally, respondents’ family size or insurance status should also be included as a factor affecting WTP.

### Willingness to Use Telehealth

Willingness to use digital tools was significantly positively associated with attitudes and self-efficacy, such as easier access to patient data (38), while WTP was likely higher due to demonstrated interest in accessing information sources (39). As demonstrated in Table 3, 132 (60.3%) out of 220 respondents were willing to use telehealth consultation services and showed a higher median WTP RM78 (JPY2,956), and those who were unwilling to use the services had a median WTP RM26 (JPY985). Respondents with a high education level and high household income showed more willingness to use telehealth consultation rather than in-person consultation in the clinic. The WTP was lower when respondents showed an unwillingness to use telehealth services. This analysis outcome was in line with the research conducted by Suzuki et al. (40), who found that respondents were willing to spend more if they had the willingness or desire to use the service. Table 3 shows that willingness to use influenced the maximum amount and that respondents were willing to pay more for telehealth consultation, which could be considered when designing an enabling market environment for increased access and use of telehealth services.

Further results from the questionnaire found that the ‘unwillingness to use’ group were not prepared to use telehealth consultation during or after the COVID-19 outbreak in Malaysia due to the lack of confidence and experience with the technology needed to communicate with healthcare providers. A similar study by Lam et al. (41) stated that the lack of readiness to use video or telephone telemedicine was primarily due to a lack of experience with the technology.

## Conclusion

In addition to the results summarised above, respondents’ sociodemographic characteristics and affordability play a critical role in their WTP decision-making process for telehealth (42), although most respondents are willing to pay for telehealth consultations. WTP for healthcare was limited by affordability and had multi factorial influences (43). Notably, those with lower income, less education, poorer health, or older age were more likely to be unwilling to use or pay because they could not afford to pay more (44). In fact, costs associated with telehealth were also identified as a barrier for telehealth service adoption (45). Hence, the cost of healthcare is an important issue from the patient’s perspective because their ability to pay may ultimately determine their healthcare choices (46). Undoubtedly, affordability is one of the key factors influencing the decision to outsource healthcare services (47).

This finding suggests that the private sector can play a crucial role in the deployment of telehealth. Governments should review existing health services (public and private) and health policymakers should consider affordability when implementing digital health services to ensure optimal access to adequate healthcare while protecting them from the financial burden that comes with it. As telehealth continues to expand, ensuring equitable healthcare accessibility by the general and vulnerable population without exacerbating disparities is a fundamental need for countries at all economic levels. Moreover, enabling Malaysia to have universal health and unlimited access to health information is also important to facilitate more widespread adoption of telehealth services.

### Limitations of the Present Study

A sampling bias is considered in this research because certain groups of citizens may not actively participate in social networks. Nevertheless, there were 27.43 million Internet users in Malaysia in January 2021, and the number of social media users is equivalent to 86.0% of the total population (48). Furthermore, the online data collection method is a limitation of this study; however, given the travel restrictions in various countries, it is currently the best way to obtain relevant data.

Furthermore, the results show that there was gender inequality in the research sample, as there were more female than male respondents. An evidence study proved that gender disparity occurs in telehealth service utilisation (49), while another review of studies demonstrated that gender differences in medical encounters may be due to differences in communication styles, perspectives and behaviours (50).

## Figures and Tables

**Figure 2:**
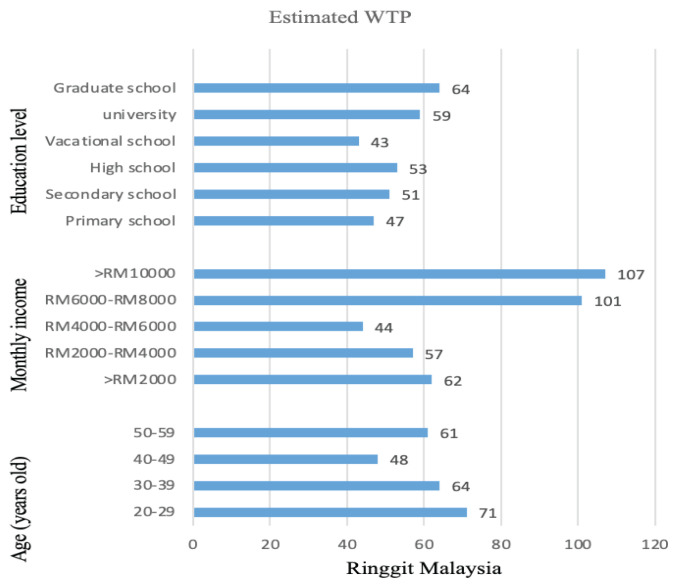
WTP estimates for the distribution of education level, age and monthly income for each group

**Table 1:** Pattern of responses

1st bid RM (JPY)	2nd bid RM (JPY)	2nd bid RM (JPY)	Y/Y (%)	Y/N (%)	N/Y (%)	N/N (%)	Total respondents
50 (1,895)	90 (3,411)	10 (379)	13 (17.8)	12 (16.4)	3 (4.1)	45 (61.6)	73
100 (3,790)	140 (5,306)	60 (2,274)	17 (22.3)	15 (19.7)	8 (0.5)	36 (47.3)	76
150 (5,685)	190 (7,201)	110 (4,169)	13 (17.1)	19 (25.0)	27 (35.5)	12 (15.7)	71
							
							220

**Table 2:** Overview of respondents’ characteristics and willingness to use telehealth consultation

Characteristics	Sample, *N* = 220 (%)	Willingness to use, *N* = 132 (%)
Gender		
Male	70 (32)	43 (61)
Female	150 (68)	89 (59)
Age in years old		
20–29	62 (28.3)	42 (68)
30–39	47 (21.5)	28 (60)
40–49	71 (32.3)	37 (52)
50–59	40 (18.2)	24 (60)
Employment status		
Employed	171 (78.1)	97 (57)
Self-employed	12 (5.5)	5 (42)
Unemployed	37 (16.9)	28 (76)
Monthly income		
Below RM2,000	60 (27.3)	39 (65)
RM2,000–RM4,000	33 (15)	19 (58)
RM4,000–RM6,000	51 (23.2)	29 (57)
RM6,000–RM9,000	66 (30)	38 (58)
RM10,000 and above	10 (4.5)	6 (60)
Highest education level		
Primary school	5 (2.3)	2 (40)
Secondary school	15 (6.8)	5 (33)
High school	9 (4.1)	5 (56)
Vocational school	2 (0.9)	1 (50)
University	175 (79.5)	110 (63)
Graduate school	14 (6.4)	8 (57)
Telehealth insurance consideration		
Yes	145 (65.9)	95 (66)
No	22 (10)	11 (50)
Undetermined	53 (24)	26 (49)

**Table 3:** Comparison of estimated WTP for respondents who were willing to use

	Willing to use RM/(JPY)	Unwilling to use RM/(JPY)	Undetermined RM/(JPY)
*n*	132	51	38
Median	78 (2,956)	26 (985)	46 (1,743)
Mean	108 (4,093)	62 (2,350 )	66 (2,401)
Average (truncated at the maximum bid)	91 (3,449)	51 (1,933)	63 (2,388)

**Highest education level**	**Willing to use ** ***n*** ** = sample**	**Unwilling to use ** ***n*** ** = sample**	**Undetermined ** ***n*** ** = sample**
Primary school	2	3	0
Secondary school	5	2	8
High school	5	3	1
Vocational school	1	0	1
University	110	338	27
Graduate school	8	4	2
Monthly income			
Below RM2,000	39	12	9
RM2,000–RM4,000	19	8	5
RM4,000–RM6,000	29	11	10
RM6,000–RM9,000	38	17	10
RM10,000 and above	6	2	2
